# CD30+ T-cell lymphoma following Chimeric Antigen Receptor T-cell therapy (CART-cell therapy): Diagnostic uncertainty in a postimmunotherapy setting

**DOI:** 10.1016/j.jdcr.2025.11.005

**Published:** 2025-11-08

**Authors:** Madison McClanahan, Madison Stokes

**Affiliations:** a College of Medicine, University of Arkansas for Medical Sciences (UAMS), Little Rock, Arkansas; b Department of Dermatology, University of Arkansas for Medical Sciences, Little Rock, Arkansas

**Keywords:** CAR-T cell therapy, CD30+ lymphoma, CD30-positive lymphoma, cutaneous T-cell lymphoma, dermatology, immune reconstitution, lymphoproliferative disorders, multiple myeloma, oncology, secondary malignancy, solitary cutaneous lesion, spontaneous regression

## Introduction

Chimeric antigen receptor (CAR) T-cell therapy is a novel immunotherapeutic approach increasingly employed in the treatment of hematologic malignancies, including B-cell leukemias, lymphomas, and multiple myeloma.^1^^,^^2^ The process involves isolation of autologous or allogeneic T cells from peripheral blood, which are genetically engineered to express synthetic CAR proteins. These modified T cells undergo clonal expansion and are reinfused into the patient following lymphodepleting chemotherapy, which enhances CAR-T cell survival and activity.^1^ Upon administration, CAR-T cells recognize tumor-associated antigens and mediate cytotoxicity through perforin and granzyme pathways.^1^

This therapy has demonstrated remarkable efficacy, even in malignancies that are refractory to conventional treatment.^3^ However, the use of CAR-T cell therapy is associated with serious adverse effects, including cytokine release syndrome, neurologic toxicity, and prolonged cytopenias.^3^ More recently, rare reports have described the emergence of T-cell lymphoproliferative disorders following this treatment.^4^, ^5^, ^6^, ^7^, ^8^ These have predominantly presented as systemic disease, whereas isolated cutaneous presentations remain uncommon. This report describes a case of biopsy-confirmed CD30-positive peripheral T-cell lymphoma with skin involvement as the solitary sight of initial presentation, arising in a patient with a recent history of B-cell maturation agent-directed CAR-T therapy. The case illustrates a variation in the usual clinical course, characterized by solitary skin involvement and regression without treatment, and raises questions about how postimmunotherapy immune modulation may influence atypical lymphoid proliferations.

## Case report

**Fig 1:**
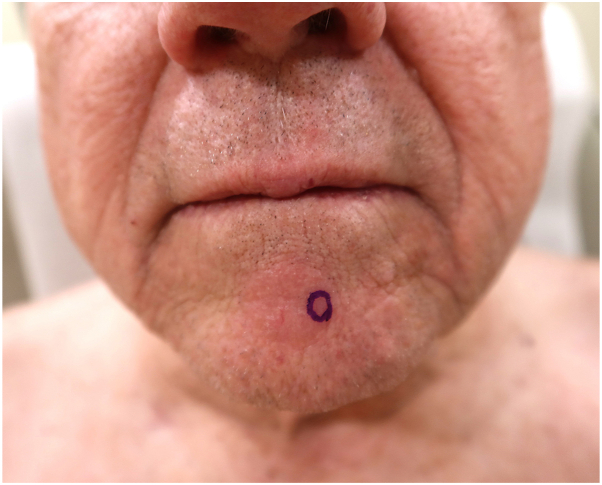
Solitary erythematous plaque on the mental crease of the chin at initial presentation. The lesion was later diagnosed as CD30-positive cutaneous T-cell lymphoma. Violet ink demarcates the punch biopsy site.

In May 2025, a 76-year-old male with a history of multiple myeloma most recently treated via B-cell maturation agent-directed ciltacabtagene autoleucel CAR-T cell therapy 9 months prior presented to the dermatology clinic with a 1-month history of a new pink plaque on the anterior chin. He denied systemic symptoms, including fever, night sweats, and weight loss. On examination, a solitary, well-demarcated, erythematous pink plaque measuring approximately 3 cm was noted on the anterior chin (Fig 1). The lesion was smooth and well circumscribed, with minimal scale and no erosions or crust. No cervical lymphadenopathy was appreciated, and laboratory values, including complete blood count and metabolic panel, were within normal limits.

**Fig 2:**
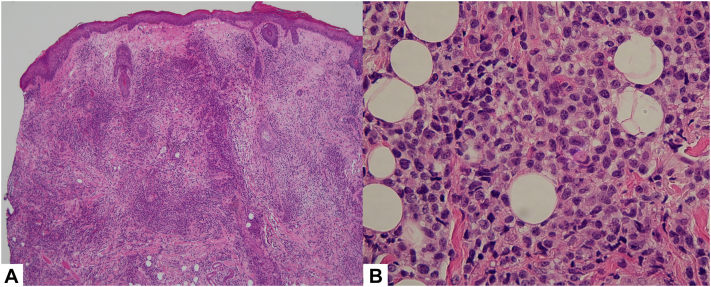
Incisional biopsy specimen (**[A]** 5× magnification, **[B]** 20× magnification) shows a dense infiltrate of atypical lymphocytes throughout the dermis, extending to the subcutaneous fat.

**Fig 3:**
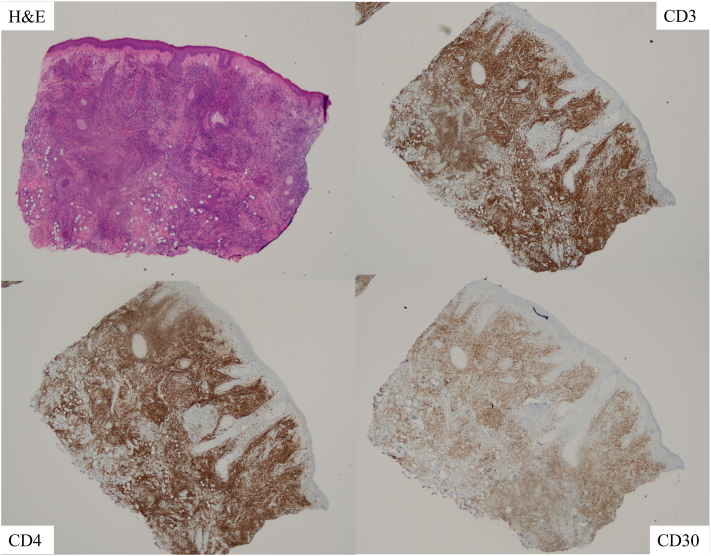
Immunohistochemical staining of the incisional biopsy shows a diffuse CD3+, CD4+, and heterogenous CD30+ T cell infiltrate.

Given the lesion’s solitary nature and nonspecific clinical features, the differential diagnosis included granuloma annulare, sarcoidosis, basal cell carcinoma, and cutaneous lymphoma. A 4-mm punch biopsy was performed, and histologic evaluation confirmed a CD30-positive cutaneous T-cell lymphoma. An incisional biopsy was then performed for diagnostic clarification (Figs 2 and 3). Genomic testing performed on an additional incisional biopsy sample confirmed a low-grade T-cell lymphoma arising from CAR-T cells.

Peripheral flow cytometry performed at the time of diagnosis showed 6% of analyzed cells to be abnormal T-cells with aberrant expression of CD30 and CD38. Positron emission tomography scans did not show any abnormal uptake, and his blood counts remained stable. His oncologist was planning to treat him with brentuximab; however, during one of their office visits, he was beginning to show signs of spontaneous resolution. At his 3-month dermatology follow-up, the plaque had completely regressed, and no new cutaneous lesions were reported. The patient remained asymptomatic otherwise, and no treatment was initiated at that time. Unfortunately, 5 months after development of the original plaque, the patient presented with a new plaque on his tongue, which was biopsy confirmed to be involved by the same T cell lymphoma. Additionally, repeat flow cytometry showed 17% of analyzed cells to be the same abnormal T cell population expressing CD30 and CD38 originally identified, consistent with progression of the peripheral T cell lymphoma with cutaneous involvement. The patient is currently being treated with brentuximab at the time of this report.

## Discussion

T-cell lymphomas occurring after CAR-T therapy remain rare, and most reported cases have involved systemic disease without cutaneous findings. This case represents an uncommon presentation of a solitary, biopsy-confirmed CD30-positive T cell lymphoma that regressed without intervention despite progression of peripheral T cell lymphoma in a patient with recent CAR-T therapy. The next-generation sequencing performed on the sample confirms that the predominant T-cell clones in this lymphoma contained CAR-T cell fusion mRNA, with potential driver mutations found in TET2, BRAF (V600E), PPM1D, ATM, and RUNX1.

The pathogenesis of secondary T-cell proliferations in the context of previous CAR-T therapy is not fully understood. Proposed mechanisms include insertional mutagenesis during CAR transduction, expansion of dormant or subclinical T-cell clones with malignant potential, or broader immune dysregulation related to lymphodepletion and immune reconstitution.^5^ Most reported cases have described systemic disease identified through blood, lymph node, or marrow involvement.^5^ In contrast, our patient presented with a solitary cutaneous lesion and no systemic findings but ultimately did have evidence of peripheral T cell lymphoma via flow cytometry.

Cutaneous involvement as the only site of disease presents unique diagnostic challenges. The lesion’s clinically ambiguous features prompted a broad differential diagnosis, increasing the potential for misdiagnosis or delay in biopsy. While reports remain rare, this case emphasizes the importance of maintaining vigilance for new lymphoproliferative processes in CAR-T therapy recipients. Dermatologists can play an important role in screening for new lymphoproliferative disorders in this patient population and should evaluate all new cutaneous lesions with high clinical suspicion. This case contributes to a growing awareness of atypical postimmunotherapy cutaneous presentations and highlights the need for further research to better understand the behavior of cutaneous T-cell proliferations and their relationship to immune reconstitution following CAR-T therapy.

## Conflicts of interest

None disclosed.
